# Quinolones and risk of haemolysis in patients with glucose-6-phosphate dehydrogenase deficiency: a nationwide retrospective active-comparator cohort study

**DOI:** 10.1093/jac/dkag265

**Published:** 2026-07-29

**Authors:** Ophir Lavon, Abdallh Khazem

**Affiliations:** Clinical Pharmacology and Toxicology Unit, Carmel Medical Center, Mikhal St. 7, Haifa 3436212, Israel; Rappaport Faculty of Medicine, Technion-Israel Institute of Technology, Haifa, Israel; Internal Medicine B, Carmel Medical Center, Haifa, Israel

## Abstract

**Background:**

Quinolone product information and avoidance lists continue to caution against use in patients with glucose-6-phosphate dehydrogenase (G6PD) deficiency despite limited comparative epidemiologic evidence. We evaluated whether oral quinolone dispensations were associated with haemolysis-related outcomes compared with an active antibiotic comparator.

**Methods:**

We conducted a nationwide retrospective active-comparator cohort study using Clalit Health Services data from 2010 to 2023. Patients with at least one ICD-coded diagnosis of G6PD deficiency who received an outpatient oral quinolone or penicillin dispensation were included. The primary outcome was a haemolysis-related diagnosis code recorded within 30 days. Jaundice and laboratory markers were secondary outcomes. Models were adjusted for age, sex and Charlson comorbidity index. Additional analyses evaluated patients aged 18 years or younger, patients exposed to both antibiotic classes and individual quinolone agents.

**Results:**

The cohort included 465 083 dispensations among 64 861 patients (65 906 quinolone and 399 177 penicillin episodes). Haemolysis-related codes were recorded after 52 quinolone episodes (0.079%) and 198 penicillin episodes (0.050%); the adjusted odds ratio (aOR) was 1.29 [95% confidence interval (CI) 0.92–1.77; *P* = 0.12]. Among patients aged 18 years or younger, events occurred after 5/8865 quinolone and 91/160 423 penicillin episodes (aOR 1.02, 95% CI 0.36–2.26, *P* = 0.970). In the within-patient subcohort, episode-level event rates were 0.065% after quinolones and 0.061% after penicillins (aOR 0.95, 95% CI 0.65–1.37, *P* = 0.785). Laboratory outcomes did not show a coherent pattern of increased haemolysis after quinolones.

**Conclusions:**

In this large outpatient cohort, oral quinolone dispensations were not associated with a detectable major increase in coded haemolysis compared with penicillins. The findings do not exclude small risks or increased susceptibility in patients with severe G6PD deficiency, very young children, active infection-related oxidative stress or concurrent oxidant exposures.

## Introduction

Glucose-6-phosphate dehydrogenase (G6PD) deficiency is the most common enzymatic disorder of erythrocytes, affecting an estimated 400 million people worldwide. The enzyme is essential for maintaining redox homeostasis in red blood cells, and deficiency predisposes to haemolytic anaemia during oxidative stress caused by selected drugs, foods or infections. Clinical expression is heterogeneous and depends on genetic variant, residual enzyme activity, age, sex and the intensity of the precipitating stressor.^1–4^

Quinolones are widely prescribed for urinary, respiratory, gastrointestinal and other bacterial infections. Historical warnings against quinolone use in G6PD deficiency are based largely on case reports of haemolysis temporally associated with ciprofloxacin, levofloxacin or ofloxacin.^5–9^ Evidence-based reviews have questioned whether therapeutic quinolone exposure produces a consistent oxidant haemolysis risk, particularly because infection itself is an important trigger and may confound drug attribution.^10^ Contemporary pharmacogenetic guidance emphasizes that medication recommendations should reflect the quality of drug-specific evidence and the severity of the G6PD phenotype.^11^

A recent large Clalit Health Services analysis evaluated acute haemolysis after exposure to multiple medications historically considered unsafe in G6PD deficiency.^12^ That study provided important system-level evidence but did not focus on the specific prescribing decision between quinolones and a common alternative antibiotic class. In addition, studies relying exclusively on diagnosis codes may be affected by outcome misclassification, recurrent coding of pre-existing disease and selective testing or coding when clinicians are aware of product-label warnings.

We therefore conducted a nationwide retrospective active-comparator study of oral quinolones versus oral penicillins and incorporated laboratory markers as supportive outcomes. The primary objective was to assess whether quinolone dispensations were associated with an increased frequency of haemolysis-related diagnosis codes within 30 days. Secondary objectives were to examine laboratory changes, paediatric patients, patients exposed to both antibiotic classes and individual quinolone agents.

## Patients and methods

### Data source and study design

We used anonymized longitudinal electronic health record data from Clalit Health Services, the largest healthcare provider in Israel, which insures more than 4.5 million individuals. Data were extracted through the MDClone platform. We conducted a retrospective active-comparator cohort study covering 1 January 2010 through 31 December 2023.

### Study population and G6PD ascertainment

Eligible participants were patients of any age with at least one recorded diagnosis code for G6PD deficiency (D55.0, anaemia due to G6PD deficiency/favism, or D75.A, G6PD deficiency without anaemia) who received an eligible antibiotic dispensation. A single qualifying code was sufficient; laboratory confirmation, quantitative enzyme activity, genotype and variant severity were not required and were not consistently available.

### Exposure definition

The unit of analysis was an outpatient pharmacy dispensation episode. Exposures were oral quinolones (ATC J01M) and oral penicillins (ATC J01C) dispensed in the community. Intravenous administrations were not included. Dose, prescribed duration, indication and adherence were not available in the analytical extract; dispensation was used as a proxy for treatment initiation. Episodes were required to be separated by at least 30 days, and episodes in which both classes were dispensed within the same 30-day risk window were excluded.

### Outcome definitions

The primary outcome was the recording of a haemolysis-related diagnosis code within 30 days after dispensation: D59.0, D59.9, D59.10 or D59.13. These codes were selected to increase the likelihood of capturing all recorded haemolytic events during the 30-day period and to reduce bias. The secondary coded clinical outcome was jaundice (R17) within 30 days. A prior haemolysis or jaundice diagnosis was not used as an exclusion criterion. Accordingly, these outcomes represent diagnosis codes recorded during the risk window and cannot be assumed to be first-ever incident events or to distinguish acute drug-induced haemolysis from recurrence or chronic haemolytic conditions.

Supportive laboratory outcomes were assessed when measurements were available. For each episode, the most recent pre-dispensation haemoglobin value and the lowest haemoglobin value within 30 days were extracted. For total bilirubin, lactate dehydrogenase (LDH), haptoglobin and reticulocyte percentage, the most abnormal value in the 30-day window was used. Pre-specified thresholds were bilirubin > 1.2 mg/dL, LDH > 300 U/L, haptoglobin < 25 mg/dL and reticulocytes > 2.5%. A categorical haemoglobin-decline threshold was not available from the extracted dataset; therefore, haemoglobin change was analysed continuously and is reported using mean, SD, median and interquartile range.

### Covariates and statistical analysis

The primary models were adjusted for age at dispensation, sex and Charlson comorbidity index (CCI).^13^ These variables were selected to represent major demographic and comorbidity differences while limiting model complexity given the small number of primary outcome events. Baseline haemoglobin was not included in the primary model because it was available only in a selectively tested subset and its inclusion would have substantially restricted the analytical cohort; baseline values were reported descriptively. The active-comparator design was intended to reduce, but could not eliminate, confounding by indication and infection severity.

Categorical variables were summarized as counts and percentages and continuous variables as means with SD and, where available, medians with interquartile ranges. Binary outcomes in the primary cohort were analysed using multivariable logistic regression; continuous haemoglobin change was analysed using linear regression. Laboratory analyses were complete-case analyses for the relevant measurement. Secondary outcomes were exploratory and no multiplicity correction was applied.

A paediatric subgroup analysis included patients aged 18 years or younger. Mixed-effects models accounted for repeated episodes and adjusted for age, sex and CCI. A within-patient sensitivity analysis included patients who received both antibiotic classes at least 30 days apart. Episode-level binary outcomes were analysed using generalized estimating equations with patient as the clustering unit. A complementary patient-level analysis used one record per patient per drug class and a binomial generalized linear mixed-effects model with a patient-level random intercept, adjusted for age and CCI at first eligible dispensation, sex and the logarithm of the total number of dispensations.

Agent-specific analyses were performed for ciprofloxacin and ofloxacin in patients who had also received penicillins. Levofloxacin and moxifloxacin were too infrequent for stable comparative models. Agent-specific models used the same patient-level mixed-effects framework.

Analyses were performed in R software (R Foundation for Statistical Computing, Vienna, Austria) using the lme4, geepack and ggplot2 packages.

### Ethics

The study was approved by the Institutional Review Board of Carmel Medical Center (approval number 151-22) and by Clalit’s national data use committee (approval number 1587).

## Results

### Primary cohort

We identified 465 083 eligible oral antibiotic dispensations among 64 861 patients with documented G6PD deficiency: 65 906 quinolone episodes among 24 710 patients and 399 177 penicillin episodes among 62 398 patients. Quinolone recipients were older and more frequently female and had higher CCI scores. Paediatric episodes represented 13.5% of quinolone and 40.2% of penicillin dispensations. Baseline characteristics are summarized in Table 1.

**Table 1.** Baseline characteristics by antibiotic group

Characteristic	Quinolones	Penicillins	*P* value
Episodes; unique patients	65 906; 24 710	399 177; 62 398	—
Age in years, mean (SD)	52.0 (26.5)	31.5 (25.9)	<0.001
Episodes in patients aged ≤18 years, *n* (%)	8865 (13.5)	160 423 (40.2)	—
Female sex, %	57	48	<0.001
CCI, mean (SD)	3.5 (3.6)	1.5 (2.4)	<0.001
Baseline haemoglobin in g/dL, mean (SD)	12.6 (1.6)	12.6 (1.6)	NS
Episodes with paired baseline/follow-up haemoglobin, *n*	49 675	231 236	—

CCI, Charlson comorbidity index; SD, standard deviation.

Haemolysis-related diagnosis codes were recorded within 30 days after 52 quinolone episodes (0.079%) and 198 penicillin episodes (0.050%). The adjusted association did not reach statistical significance [adjusted odds ratio (aOR) 1.29, 95% confidence interval (CI) 0.92–1.77; *P* = 0.12]. The absolute difference was 0.029%, equivalent to approximately 3 additional coded events per 10 000 dispensations if the association were causal. At the patient level, at least one coded event occurred in 45/24 710 quinolone-exposed patients (0.18%) and 120/62 398 penicillin-exposed patients (0.19%; aOR 0.784, 95% CI 0.544–1.111; *P* = 0.18).

Jaundice codes were more frequent after quinolone episodes (68/65,906, 0.103%) than after penicillin episodes (103/399,177, 0.026%; aOR 3.07, 95% CI 2.19–4.79; *P* < 0.0001), although the absolute excess was approximately 0.07%. Because R17 is non-specific and prior jaundice diagnoses were not excluded, this finding was interpreted with the laboratory outcomes.

Among episodes with paired haemoglobin measurements, mean decline was 1.7 (SD 1.9) g/dL after quinolones and 1.2 (SD 1.8) g/dL after penicillins; median declines were 1.3 (IQR 0.4–2.6) and 0.8 (IQR 0.1–1.9) g/dL, respectively. The adjusted between-group difference was 0.068 g/dL (95% CI 0.051–0.085; *P* < 0.0001). Bilirubin elevation, low haptoglobin and reticulocytosis were not significantly more frequent after quinolones; LDH elevation was slightly less frequent. The laboratory results did not show a coherent biochemical pattern of increased haemolysis (Table 2).

**Table 2.** Outcomes within 30 days after antibiotic dispensation in the primary cohort

Outcome	Quinolones	Penicillins	Adjusted effect (95% CI)	*P* value
Haemolysis-related diagnosis code, *n*/*N* (%)	52/65 906 (0.079)	198/399 177 (0.050)	OR 1.29 (0.92–1.77)	0.12
Patients with ≥1 haemolysis-related code, *n*/*N* (%)	45/24 710 (0.18)	120/62 398 (0.19)	OR 0.784 (0.544–1.111)	0.18
Jaundice code, *n*/*N* (%)	68/65 906 (0.103)	103/399 177 (0.026)	OR 3.07 (2.19–4.79)	<0.0001
Bilirubin > 1.2 mg/dL, *n*/*N* (%)	1247/10 519 (11.9)	3369/29 943 (11.3)	OR 1.057 (0.983–1.137)	0.13
LDH > 300 U/L, *n*/*N* (%)	6148/8564 (71.8)	18 098/24 366 (74.3)	OR 0.854 (0.798–0.896)	<0.0001
Haptoglobin < 25 mg/dL, *n*/*N* (%)	5/243 (2.1)	21/591 (3.6)	OR 0.67 (0.21–1.73)	0.44
Reticulocytes > 2.5%, *n*/*N* (%)	84/183 (45.9)	378/971 (38.9)	OR 1.038 (0.723–1.478)	0.83
Haemoglobin decline, mean (SD), g/dL	1.7 (1.9)	1.2 (1.8)	Adjusted beta 0.068 (0.051–0.085)	<0.0001

SD, standard deviation.

### Within-patient sensitivity analysis

The within-patient subcohort included 22 247 patients with 59 889 quinolone and 173 983 penicillin episodes separated by at least 30 days. Haemolysis-related codes occurred after 39 quinolone episodes (0.065%) and 106 penicillin episodes (0.061%); the adjusted episode-level estimate was 0.95 (95% CI 0.65–1.37; *P* = 0.785). Jaundice codes and bilirubin above 1.2 mg/dL were somewhat more frequent after quinolones, whereas LDH elevation was less frequent and no material differences were observed for haptoglobin or reticulocytes (Table 3).

**Table 3.** Within-patient analysis among patients exposed to both antibiotic classes

Outcome	Quinolone episodes	Penicillin episodes	Adjusted effect (95% CI)	*P* value
Episodes, *n*	59 889	173 983	—	—
Haemolysis-related diagnosis code, *n*/*N* (%)	39/59 889 (0.065)	106/173 983 (0.061)	OR 0.949 (0.647–1.366)	0.785
Jaundice code, *n*/*N* (%)	59/59 889 (0.099)	48/173 983 (0.028)	OR 2.931 (1.990–4.337)	<0.0001
Bilirubin > 1.2 mg/dL, *n*/*N* (%)	1119/9485 (11.8)	1894/18 090 (10.5)	OR 1.129 (1.042–1.223)	0.003
LDH > 300 U/L, *n*/*N* (%)	5447/7667 (71.0)	11 096/14 966 (74.1)	OR 0.816 (0.766–0.869)	<0.0001
Haptoglobin < 25 mg/dL, *n*/*N* (%)	5/206 (2.4)	7/365 (1.9)	OR 1.282 (0.372–4.106)	0.677
Reticulocytes > 2.5%, *n*/*N* (%)	46/105 (43.8)	73/186 (39.2)	OR 1.225 (0.739–2.031)	0.430
Haemoglobin decline, mean (SD), g/dL	1.7 (1.9)	1.5 (1.8)	Adjusted beta −0.029 (−0.049 to −0.010)	0.003

SD, standard deviation.

In the complementary patient-level analysis, 35/22 247 patients had a haemolysis-related code after quinolone exposure and 57/22 247 after penicillin exposure. The adjusted mixed-effects estimate did not demonstrate an increase after quinolones (aOR 0.40, 95% CI 0.12–1.30; *P* = 0.127).

### Paediatric subgroup

The paediatric analysis included 169 288 episodes among 27 572 patients aged 18 years or younger: 8865 quinolone and 160 423 penicillin episodes (Table 4). Haemolysis-related codes were recorded after 5 quinolone episodes (0.056%) and 91 penicillin episodes (0.057%); the aOR was 1.02 (95% CI 0.36–2.26; *P* = 0.970). Haemolysis-related codes were recorded for 55/25 239 paediatric patients exposed to penicillins and 5/4829 for quinolone exposure; the aOR was 1.7 (0.56–4.21, *P* = 0.292). There was no significant increase in jaundice, bilirubin elevation, haptoglobin reduction or reticulocytosis. LDH elevation was less frequent after quinolones. The adjusted difference in haemoglobin decline was −0.074 g/dL (95% CI −0.128 to −0.019; *P* = 0.008), a clinically negligible magnitude. In the subgroup of children exposed to both classes, the aOR for an episode-level haemolysis-related code was 1.08 (95% CI 0.36–2.66; *P* = 0.871).

**Table 4.** Paediatric subgroup (aged ≤18 years)

Outcome	Quinolones	Penicillins	Adjusted effect (95% CI)	*P* value
Episodes, *n*	8865	160 423	—	—
Haemolysis-related diagnosis code, *n*/*N* (%)	5/8865 (0.056)	91/160 423 (0.057)	OR 1.017 (0.358–2.257)	0.970
Jaundice code, *n*/*N* (%)	1/8865 (0.011)	41/160 423 (0.026)	OR 0.445 (0.025–2.041)	0.423
Bilirubin > 1.2 mg/dL, *n*/*N* (%)	27/347 (7.8)	396/5667 (7.0)	OR 1.033 (0.668–1.536)	0.879
LDH > 300 U/L, *n*/*N* (%)	221/264 (83.7)	4085/4426 (92.3)	OR 0.473 (0.329–0.692)	<0.001
Haptoglobin < 25 mg/dL, *n*/*N* (%)	0/7 (0)	5/75 (6.7)	Not estimable	—
Reticulocytes > 2.5%, *n*/*N* (%)	10/20 (50.0)	211/610 (34.6)	OR 2.038 (0.808–5.154)	0.127
Haemoglobin decline, mean (SD), g/dL	0.3 (1.6)	0.3 (1.6)	Adjusted beta −0.074 (−0.128 to −0.019)	0.008
Patients with at least one haemolysis-related diagnosis code, *n*/*N* (%)	5/4829 (0.2)	55/25 239 (0.1)	OR 1.7 (0.56–4.21)	0.292

SD, standard deviation.

### Individual quinolone agents

Agent-level information was available for 65 903 of 65 906 quinolone episodes. Ciprofloxacin accounted for 36 620 episodes (55.6%), ofloxacin for 29 193 (44.3%), levofloxacin for 84 (0.13%) and moxifloxacin for 6 (0.01%). No norfloxacin or nalidixic acid episodes were identified in the supplied agent-level extract. In patients exposed to both ciprofloxacin and penicillins, haemolysis-related codes occurred in 25/13 606 after ciprofloxacin and 37/13 606 after penicillins (aOR 0.45, 95% CI 0.13–1.10; *P* = 0.23). For ofloxacin, events occurred in 4/7421 after ofloxacin and 29/7421 after penicillins (aOR 0.001, 95% CI 0.0001–19.01; *P* = 0.21). The adjusted model for ofloxacin should be interpreted with caution as it is based on sparse events and may not provide a reliable effect estimate.

## Discussion

In this nationwide outpatient active-comparator cohort, oral quinolone dispensations were not associated with a statistically significant increase in haemolysis-related diagnosis codes compared with penicillins. The absolute frequency of coded events was below 1 per 1000 episodes in both groups. Additional analyses in paediatric patients, patients exposed to both antibiotic classes and the predominant individual agents did not identify a major haemolysis signal. These findings should be interpreted as an absence of a detectable population-level signal rather than proof that quinolones cannot precipitate haemolysis in any susceptible individual.

The present study should be interpreted in the context of the previously published analysis by Gronich *et al*., which evaluated medication-associated acute haemolysis among G6PD-deficient members of the same Israeli healthcare system.^12^ Although the two studies drew from an overlapping source population, they addressed the question using materially different designs and ascertainment strategies. Gronich *et al*. used a case-control approach and identified G6PD deficiency primarily through laboratory testing, whereas the present study used an active-comparator cohort design, identified patients through diagnostic coding and focused specifically on the clinically relevant comparison between quinolone and penicillin dispensations. The current analysis also incorporated episode-level risk windows, laboratory markers, paediatric and individual-agent analyses and a within-patient comparison. The use of different designs and different methods of identifying G6PD deficiency creates an opportunity for methodological triangulation. From a pharmacovigilance perspective, concordant findings across a case-control study based on laboratory-confirmed G6PD deficiency and an active-comparator cohort based on diagnostic coding provide greater reassurance than either study alone, because the conclusion is less likely to be explained by a single design-specific source of bias.

The increased frequency of coded jaundice requires a balanced interpretation. Jaundice is non-specific and may reflect hepatobiliary disease, infection severity, recurrent coding or differential evaluation after a drug carrying a warning. In the within-patient analysis, bilirubin above 1.2 mg/dL was also modestly more frequent after quinolones, but this was not accompanied by more coded haemolysis, greater adjusted haemoglobin decline, increased LDH, reduced haptoglobin or a clear reticulocyte response. The discordant pattern does not support a coherent syndrome of clinically important oxidant haemolysis, although selective laboratory testing and incomplete phenotype information limit certainty.

The paediatric findings are clinically relevant because children with G6PD deficiency may experience abrupt and severe haemolytic crises after oxidative triggers, and the clinical significance of a haemoglobin decrease depends on age, baseline haemoglobin, rate of decline and compensatory response.^14,15^ In the present cohort, quinolone use in children was uncommon relative to penicillin use, and only five coded events followed quinolone dispensations. The point estimate was close to unity, but CIs were wide; therefore, the paediatric analysis is reassuring only in the limited sense that no large increase was detected. It cannot exclude uncommon risk in neonates, very young children or children with severe enzymatic deficiency.

The agent distribution further limits generalization. Nearly all quinolone episodes were ciprofloxacin or ofloxacin. The ciprofloxacin analysis did not identify an increased risk, and the ofloxacin event count was low, but the ofloxacin model was statistically unstable. There were too few levofloxacin and moxifloxacin episodes for meaningful estimates and no nalidixic acid exposure in the supplied extract. Consequently, class-level findings should not be extrapolated with equal confidence to rarely used agents or older non-fluorinated quinolones.

The lack of quantitative enzyme activity and genotype data is an important limitation. Requiring only one diagnosis code may include misclassified patients, and non-differential misclassification could bias estimates towards no association. Conversely, if the coded cohort predominantly represents patients with milder variants or higher residual enzyme activity, the observed results could overstate safety for patients with severe deficiency. The within-patient analysis controls for stable patient characteristics, including genotype and baseline phenotype, but does not correct diagnostic misclassification, time-varying infection severity or differences in concurrent oxidant exposures.

From an antimicrobial-stewardship perspective, the findings do not justify broader quinolone prescribing or override established indications, resistance patterns and class-specific adverse effects. They do suggest that a G6PD diagnosis alone should not automatically preclude an otherwise appropriate oral quinolone when alternatives are unsuitable. Decisions should remain individualized and should consider infection severity, previous haemolytic history, known severe G6PD deficiency, age, baseline anaemia, renal and hepatic function and concomitant oxidant drugs.

Other limitations of the study are inherent to retrospective database research. Pharmacy dispensation was used as a proxy for treatment initiation and does not confirm ingestion, adherence, dose or treatment duration. Although all exposures were outpatient dispensations of oral antibiotics, residual confounding by indication and infection-related oxidative stress remains possible because information on infection site and severity, inflammatory status, concurrent oxidant exposures and other time-varying clinical factors was unavailable. Laboratory testing was performed selectively in routine care, creating potential surveillance bias and limiting laboratory analyses to non-random subsets of episodes. A categorical, clinically defined haemoglobin-decline endpoint could not be reconstructed, and data on transfusion requirements and exact time to event were unavailable. Although repeated episodes and within-patient correlation were addressed using clustered and mixed-effects analyses, these approaches cannot eliminate time-varying confounding or coding error. Finally, secondary outcomes and subgroup analyses were exploratory and were not adjusted for multiple comparisons.

Study strengths include the nationwide sample, active-comparator design, outpatient oral exposure definition, large paediatric subgroup, laboratory triangulation and analyses restricted to patients who received both antibiotic classes.

Pending further evidence, clinicians should maintain enhanced vigilance in very young children, patients with known severe G6PD deficiency or previous haemolytic crises, patients with active infection-related oxidative stress and those receiving multiple potentially oxidant medications.

### Conclusions

In this large nationwide cohort of patients with coded G6PD deficiency, outpatient oral quinolone dispensations were not associated with a detectable major increase in coded haemolysis compared with oral penicillins. Paediatric, within-patient and ciprofloxacin analyses were directionally consistent with the primary finding, but sparse events and incomplete phenotype and clinical data preclude an inference of complete safety. The results support individualized prescribing and vigilance rather than automatic avoidance of all quinolones solely based on a G6PD diagnosis.

## Data Availability

The data that support the findings of this study are available from the corresponding author upon reasonable request and subject to Clalit Health Services data-governance requirements.
